# Effect of roughness and acidic medium on wear behavior of dental resin composite

**DOI:** 10.1186/s12903-022-02516-4

**Published:** 2022-11-05

**Authors:** Jiawen Guo, Zhaoxia Bing, Jiajun Yang, James K. H. Tsoi, Yan Wang

**Affiliations:** 1 Department of Prosthodontics, Hospital of Stomatology, Guangdong Provincial Key Laboratory of Stomatology, Guanghua School of Stomatology, Sun Yat-sen University, 56 Lingyuan Xi Road, Guangzhou, 510055 China; 2 Dental Materials Science, Division of Applied Oral Sciences and Community Dental Care, Faculty of Dentistry, The University of Hong Kong, 34 Hospital Road, Sai Ying Pun, Hong Kong SAR, China

**Keywords:** Resin composite, Surface roughness, Wear, Lubrication, Citric acid

## Abstract

**Background:**

The aim of the study was to investigate whether the citric acid and rough surface have a synergistic effect leading to severe wear behavior of resin composite.

**Materials and methods:**

Disk-shaped (Ø15 × 1.5 mm) specimens of resin composite (*n* = 12) with different initial roughness were prepared. Reciprocating ball-on-flat wear tests were performed under distilled water and citric acid (pH = 5.5) respectively. The coefficient of friction (COF), wear volume loss, and duration of the running-in period were quantified to assess the wear performance. And the values were analyzed with one-way ANOVA (α = 0.05). Regression analysis was applied to examine the influence of Ra values and mediums on the wear data. The wear morphology was analyzed by scanning electron microscopy and a 3D profilometer.

**Results:**

The average COF was higher in distilled water than in citric acid but was independent of the surface roughness. For the composite, the volume loss of worn area and running-in period increased with surface roughness when tested under distilled water. However, these increasing trends were not found in citric acid. All specimens exhibited mild wear behavior with low COF and less superficial abrasion in acidic medium.

**Conclusions:**

The effect of initial roughness on wear behavior depends on the medium. In distilled water, resin composites with high initial roughness exhibit a longer running-in time, which eventually leads to a significant increase in material loss. The adverse effects of high roughness can be alleviated by the lubrication of citric acid, which can maintain a mild wear behavior regardless of initial surface roughness.

## Background

Resin composites are widely used in dentistry to restore cavities caused by caries. Several advantages, such as good esthetics, accepted fracture resistance, ability to bond to tooth structures, and ease of handling, make it an excellent candidate to replace amalgam fillings. However, excessive wear of the resin composite restoration, which will lead to premature fracture [1] and material loss of occlusal surface, remains a major clinical problem. Particularly in patients who have bruxing and clenching habits, a high magnitude of lateral contact stress during chewing contributes to the high failure rate of posterior restorations [2, 3]. Therefore, it is essential to evaluate the wear behavior of the resin composite in vitro for understanding its clinical performance.

Friction and wear of resin composite not only depend on its composition, such as the size, distribution, and content of inorganic fillers [4, 5], but also on the external factors, including (1) the interaction conditions (loading [6] and duration of interaction [7]); (2) microenvironment (temperature [8], pH value [9], humidity [10]); (3) surface condition of materials (surface topography) [11, 12]. As a bio-tribological system, wear behavior in the oral cavity can be affected mainly by masticatory force, food bolus, lubricating medium, and surface roughness of two contacting bodies [13–15].

With the prevalent consumption of carbonated beverages and juices which contain citric acid, the dental composite restorations are more frequent subjected to acid challenge [16]. It has been reported that the surface hardness of dental composite decreased significantly owing to hydrolyzed effect of acid, which result in a softened surface [17, 18]. Theoretically, this weakened superficial layer can be easily worn by the antagonist during mastication. This in turn further decrease the wear resistance of resin composite. But some in vitro studies [19, 20] revealed that the carboxylic acid exhibited an excellent lubricating property by forming a boundary lubrication layer between restorative materials and its antagonist, which could reduce the wear loss and friction coefficient. Yu Ping et al. [21] found that a polymer infiltrated ceramic network (PICN) had a lower coefficient of friction (COF) and wear loss in lactic acid than in artificial saliva. Also, a study showed that the wear loss of veneered lithium disilicate tested in artificial saliva was 1.6 times higher than in citric acid [22]. During the mastication process, whether the citric acid acts as a lubricating medium or serves as an erosive agent remains unclear.

Meanwhile in oral environment, the surface roughness of dental restoration may increase with time due to erosive damage and abrasive effect [23, 24]. A rough surface adversely affects wear behavior of both dental restoration and its antagonistic natural teeth [25]. According to tribology, the topography of the interacting surfaces may regulate the wear process by influencing the contact and friction conditions. For the contact surface with high roughness value, wear behavior tends to be aggravated by the small contact area and high contact pressure [26]. In fact, composite restorations would exhibit distinguished surface roughness depending on patients’ individual circumstance, thus demonstrating different wear process.

Acidic diet and surface roughness as mentioned above are critical to wear behavior of dental restoration, which, however, are commonly studied in isolation. In the oral cavity, multiple challenging factors may be present at the same time. Whether the citric acid solution and rough surface contribute synergistically to a sever wear process, or the adverse effect of rough surface on wear process can be offset by the lubrication of citric acid medium is unknown. Therefore, the aim of this study was to investigate the relationship between citric acid medium and surface roughness on friction and wear of resin composite. Twelve specimens with different surface roughness were prepared. Reciprocating ball-on-flat wear tests were performed under two mediums (distilled water and citric acid) to examine the coefficient of friction, wear volume loss, and the morphology of worn surfaces. The null hypothesis was that the wear behaviors of resin composites with different surface roughness were similar no matter measured in citric acid medium or distilled water.

## Material and methods

### Preparation of resin composite samples

A conventional resin composite (Z350, Filtek™ Z350XT; 3 M ESPE, USA), shade A2, was used in this study. Its characteristic features are shown in Table 1. Twelve disc-shaped specimens (diameter 15 mm × thickness 1.5 mm) were obtained by using a Teflon mold. The composite material was filled into the cavity of mold incrementally to avoid bubbles, then the molds were pressed with a glass slide. The discs were light polymerized for 20 seconds using a light-emitting diode unit (LED, S-10TM, 3 M ESPE, St Paul, USA). Each disc was then embedded in self-curing epoxy resins (Mucklin, Shanghai, China) using a silicone mold (20 × 20 × 20 mm) with the flat surface of resin composite exposed.

**Table 1:** Product information of composites used in the study

Material	Type	Organic matrix	Inorganic filler	Filler load% weight (volume)	Manufacture	Lot
Filtek™ Z350 XT	Nanofilled composite	Bis-GMA, Bis-EMA, TEGDMA, UDMA, PEGDMA	Silica, Zirconia, and aggregated zirconia/silica clusters	72.5 (63.3)	3 M-ESPE, St. Paul, MN, USA	NE65754

*Bis-GMA* bisphenol-A-glycidyl dimethacrylate, *Bis-EMA* ethoxylated bisphenol-A-dimethacrylate, *TEGDMA* triethylene glycol dimethacrylate, *UDMA* urethane dimethacrylate, *PEGDMA* Poly (ethylene glycol) dimethacrylate

### Grinding and polishing

To obtain the resin composites with different surface roughness, specimens were subjected to different grinding procedures. In order to get a smooth surface, the specimen was ground and polished by a series of sandpaper (− 200, − 400, − 800, − 1200, − 2000, − 3000 grit). While for a rough surface, the samples were only ground by sandpaper with 200 grits. The detailed induvial grinding procedures for each specimen are summarized in Table 2. All the grinding and polishing procedures were performed on a polishing machine (UNIPOL-1502, Shenyang Kejing Auto-Instrument Co., China) under permanent water cooling, 5 N load, 125 rpm rotational speed. All the procedures were performed by a single trained investigator. Finally, all specimens were ultrasonically cleaned for 10 min in deionized water.

**Table 2:** Grinding procedures and surface roughness (Ra, mean ± standard deviation) of twelve resin composites

Grinding procedure	Sample code	Ra (μm)
200 grit	a	2.03 ± 0.63
	b	1.60 ± 0.23
200, 400 grit	c	0.61 ± 0.04
	d	0.50 ± 0.04
200, 400, 800 grit	e	0.78 ± 0.05
	f	0.58 ± 0.04
200, 400, 800,1200 grit	g	0.42 ± 0.08
	h	0.45 ± 0.02
200, 400, 800,1200,2000 grit	i	0.14 ± 0.02
	j	0.19 ± 0.03
200, 400, 800,1200,2000, 3000 grit	k	0.06 ± 0.01
	l	0.05 ± 0.004

The surface roughness of each specimen was measured with a white light scanning profilometer (MFT-3000, Rtec, USA) before the wear tests. The surface roughness was characterized by the height parameter Ra (μm), which represents the arithmetic mean of the profile deviation. 3D surface topographies and profile roughness were evaluated using the commercial software (Gwyddion 2.30, Czech Metrology Institute, Czech Republic). Three successive measurements were recorded for each sample, and the average surface roughness (Ra) was obtained.

### Wear testing

Two solutions were used in the wear test. Citric acid solution with pH 5.5, which is commonly seen in oral environment after consumption of beverages and fruits, was selected as the representative acidic condition. And distilled water was used as a control. The pH value of the distilled water was 7.05 ± 0.03 immediately after distillation, and decreased to 6.45 ± 0.09 after 30 min wear testing due to absorption of CO_2_ when exposed to air. The concentration of the citric acid solution was 0.26 mol/L, which gave a pH value of 1.8 naturally. NaOH (Sigma-Aldrich, Shanghai, China) was added to the original citric acid solution till pH reached 5.5.

Reciprocating wear tests were conducted on a tribometer, using a ball-on-flat contact mode (HSR-2 M, Lanzhou Zhongkekaihua, China). Before the wear testing, the disk-shaped specimen was fixed to the bottom of a stainless-steel reservoir that contained the fluid (distilled water or citric acid). Zirconia balls with a diameter of 4 mm were used as the antagonist. The test was performed under a normal load of 5 N at a frequency of 5 Hz with a total stroke length of 3 mm for 30 min. The COF was recorded. After wear testing, specimens were rinsed with distilled water immediately to remove any residual medium. For each specimen, three tests were performed for each medium. A fresh solution was used for each test to ensure consistency and stability. The distance between two adjacent wear scars was at least 1.5 mm. All experiments were performed at room temperature.

### Wear loss

Following wear testing, the dimension of the wear scar was measured by a white light scanning profilometry (MFT-3000, Rtec, USA). The scanned area covered entire wear scar. A reference plane for the dimension measurement was defined by selecting three points on the unworn surface. And the volume loss of specimen was calculated using dedicated software. Three-dimensional topography maps of the wear tracks were reconstructed using the commercial software (Gwyddion 2.30, Czech Metrology Institute, Czech Republic). Wear scar profiles were selected randomly at the middle of the abrasion region perpendicular to the sliding direction. Then depth and width of the wear scar were measured from these profiles.

### Morphology observation

Surface characterization and wear pattern were evaluated by scanning electron microscopy (SEM, Sigma 300, Zeiss, Germany). The morphologies of the worn surfaces were analyzed to identify the wear mechanisms in different lubricants. Specimen was cleaned in an ultrasonic bath with distilled water for 5 min and then air-dried. The surface was gold-sputtered and analyzed by SEM under 100 and 5000 magnifications to assess the surface characteristics.

### Running-in time

The duration of running-in stage for each specimen was measured by the reciprocating wear tests with a low load and frequency (1 N and 0.5 Hz). In this way, the details of the running-in stage can be demonstrated more clearly, and thus provide an opportunity to gain a better understanding of the effect of roughness and lubricant on the running-in stage. Other parameters of the wear test were similar to Wear testing section.

### Statistical analysis

SPSS statistical software (SPSS Statistics Version 25.0, IBM Corporation, USA) was used to perform the descriptive analysis of the COF, roughness (Ra), and the volume loss to obtain the mean and standard deviation values. The normality of data distribution was tested using the Shapiro–Wilk test. One–way ANOVA was performed to assess the differences in COF and wear volume loss of resin composites in different mediums. A *p*-value of less than 0.05 was considered statistically significant. Regression analysis was applied to examine the influence of Ra values and mediums on the wear data (COF, volume loss, and running-in time).

## Results

### Surface roughness and 3D morphology

**Fig. 1:**
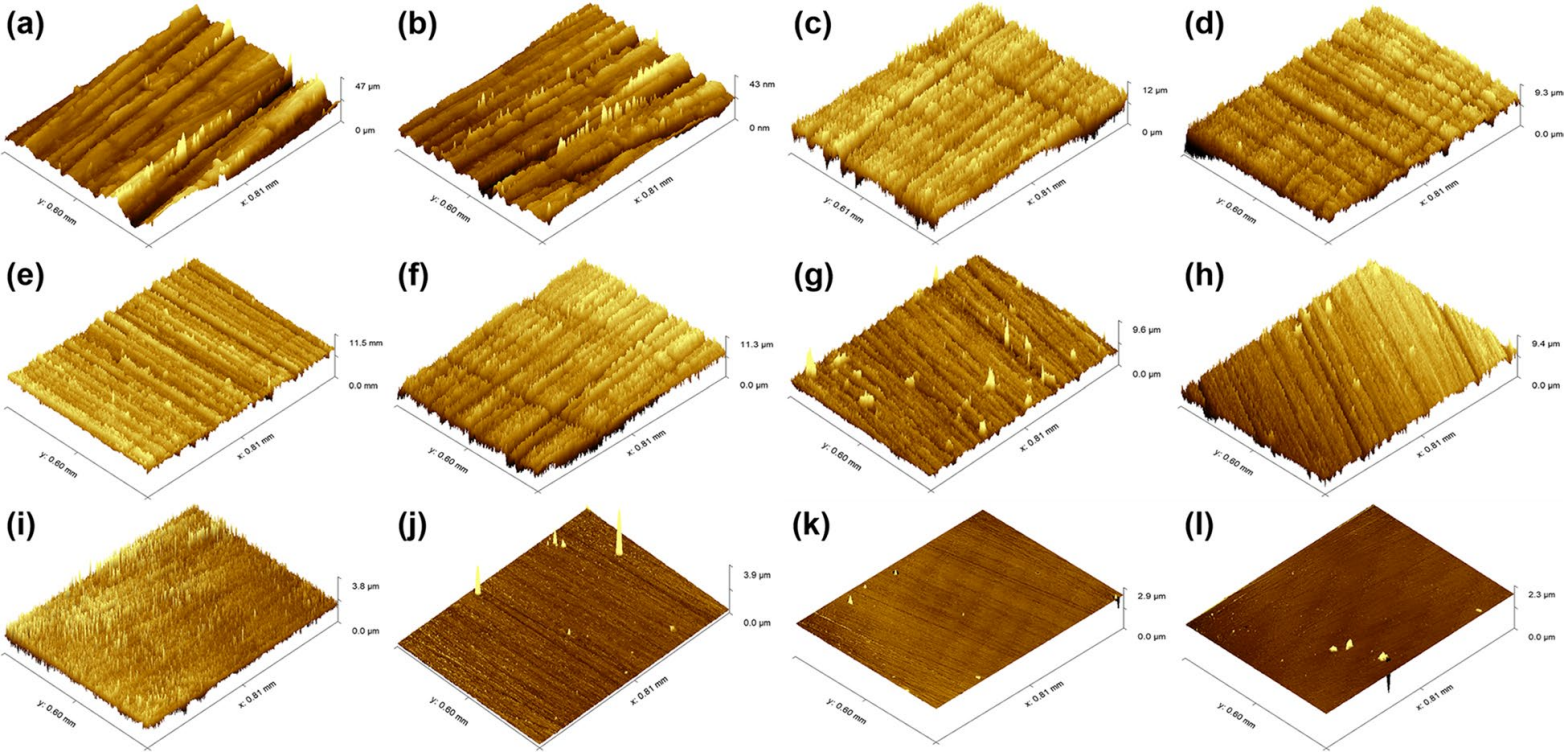
3D topographies of all resin composites with different Ra: **a**: 2.03 ± 0.63 μm; **b** 1.60 ± 0.23 μm; **c** 0.61 ± 0.04 μm; **d** 0.50 ± 0.04 μm; **e** 0.78 ± 0.05 μm; **f** 0.58 ± 0.04 μm; **g** 0.42 ± 0.08 μm; **h** 0.45 ± 0.02 μm; **i** 0.14 ± 0.02 μm; **j** 0.19 ± 0.03 μm; **k** 0.06 ± 0.01 μm; **l** 0.05 ± 0.004 μm

Table 2 shows the surface roughness (Ra) of each specimen, ranging from 0.05 ± 0.004 to 2.03 ± 0.63 μm. The typical 3D topographies of 12 specimens with different Ra are illustrated in Fig. 1. Deep unidirectional scratches caused by grinding can be observed on the rough surface (Fig. 1a, b). Scratches became shallow and narrow when Ra decreased to 0.78 μm and below (Fig. 1c-h). And specimens with smooth surfaces had little or no scratches, as shown in Fig. 1i-l.

### Coefficient of friction (COF)

**Fig. 2:**
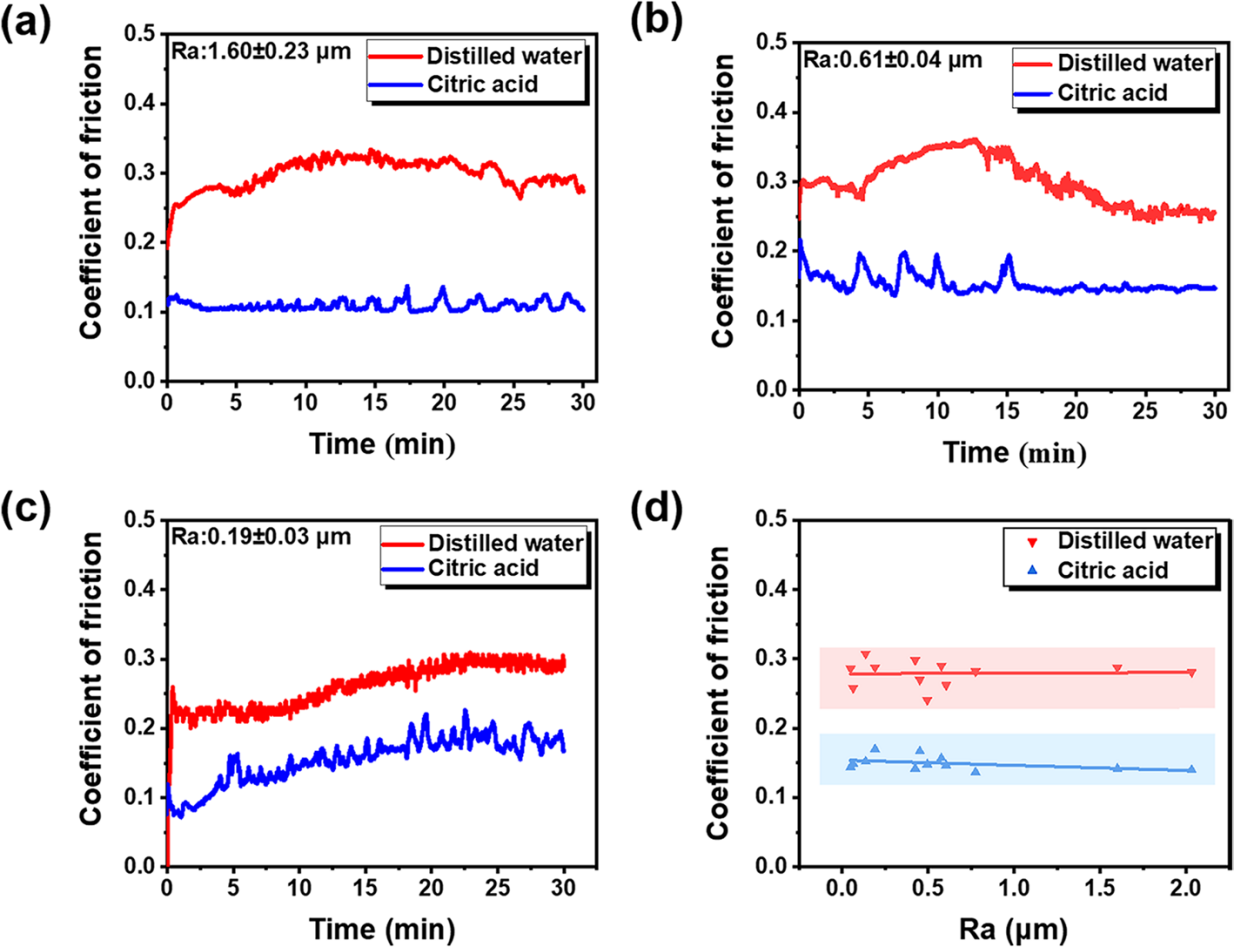
The COF curves for the resin composite with different Ra in distilled water and citric acid: **a** 1.60 ± 0.23 μm; **b** 0.61 ± 0.04 μm; **c** 0.19 ± 0.03 μm. **d** Average COF as a function of Ra in distilled water and citric acid. The regression lines represent the best fit to the experimental data

The COF curves of three representative specimens with different Ra in two mediums are depicted in Fig. 2. The development of the COF for each specimen could be roughly divided into two stages, including the running-in stage, in which the friction coefficient increases rapidly, and the steady stage, where the friction coefficient remains stable (Fig. 2a-c). The average COF values for each testing condition were determined by calculating the arithmetic mean of the COF during the steady-state stage. With the same medium, specimens showed similar average COF, regardless of the initial roughness. Regression lines for average COF vs. Ra values in two mediums are presented in Fig. 2d. The linear regression slope coefficients, standard errors, and *P*-values of the linear regression model are summarized in Table 3. Linear regression analysis of variance showed that the Ra values had no influence on the average COF, no matter in distilled water or citric acid (*P* > 0.05). Moreover, One–way ANOVA test revealed that the average COF in citric acid (0.15 ± 0.01) was significantly lower than that in distilled water (0.28 ± 0.02) (*P*<0.001).

**Table 3:** Linear regression slope coefficients, standard errors (SE) and F, *P*-values of the linear regression model for COF in each lubricant

Lubricant	Regression slope (SE)	F	*P*
Distilled water	0.005(0.004)	2.416	0.151
Citric acid	−1.02 (0.011)	0.01	0.922

### Wear profile and wear loss

**Fig. 3:**
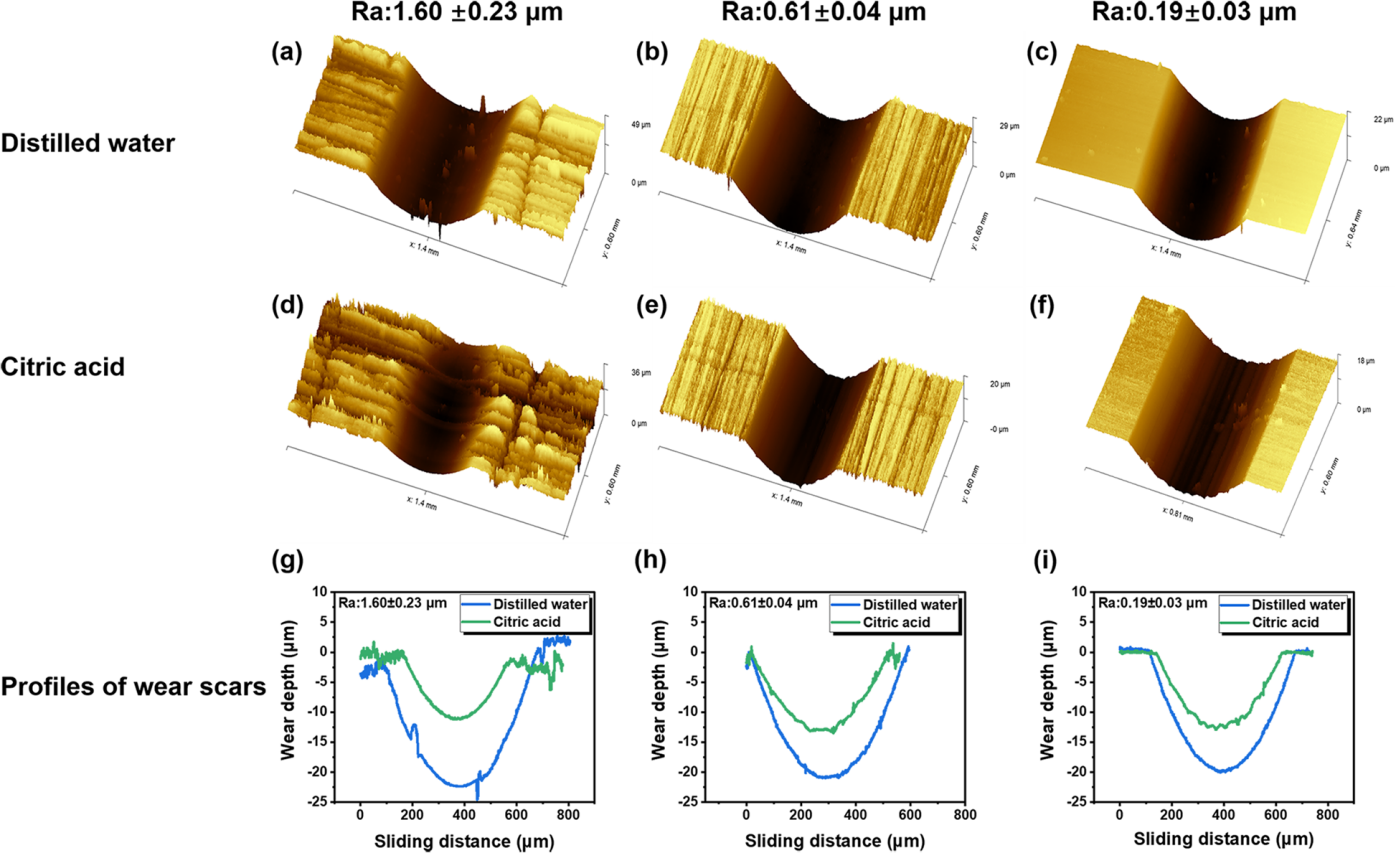
Representative 3D topographies (**a-f**) and profiles of wear scars (**g-i**) of resin composite with different Ra in two lubricants

3D topographic images and profiles of wear tracks for three representative specimens are presented in Fig. 3. Compared to the citric acid group, the specimen in distilled water exhibited greater wear depth and width regardless of roughness (Fig. 3a-f). For the disc with the Ra of 0.19 μm, the width of wear scar increased from 487.4 to 574.3 μm and the depth increased from 12.5 to 20.2 μm when the medium changed from citric acid to distilled water (Fig. 3i). Moreover, in distilled water, the wear depth and width of the resin composite showed an increasing trend with its surface roughness. As the Ra value increased from 0.19 to 1.6 μm, the wear width and depth of specimen in distilled water increased to 637.5 and 24.3 μm, respectively (Fig. 3g-i). However, the dimension of the wear scar changed slightly with the increasing of roughness when tested in acidic solutions.

**Fig. 4:**
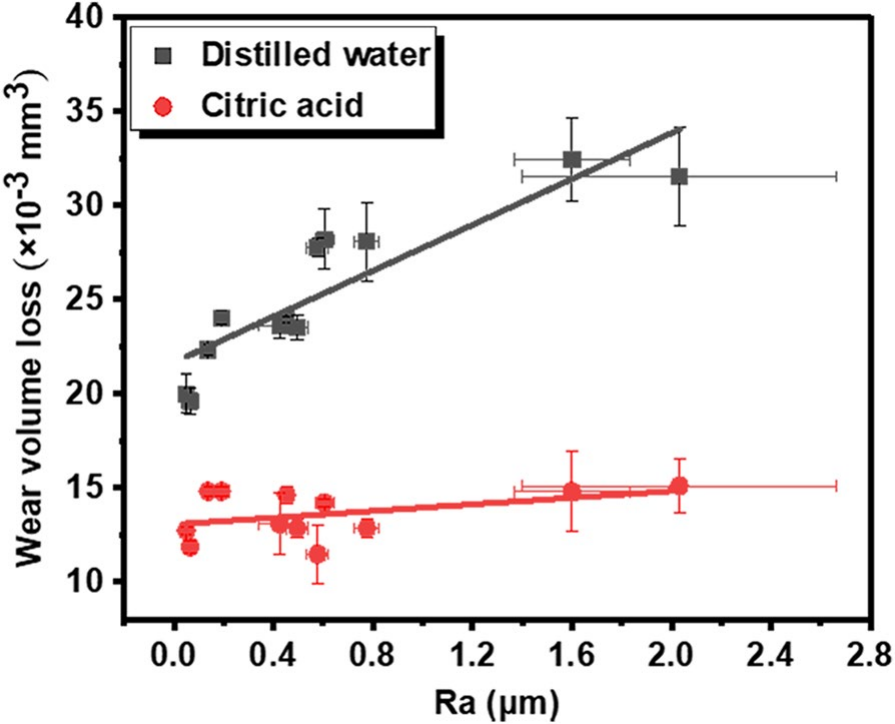
Wear volume loss of resin composite as function of Ra in distilled water and citric acid. The regression lines represent the best fit to the experimental data

The average wear loss of resin composite in citric acid (1.362 ± 0.127 × 10^− 2^ mm^3^) was significantly lower comparing to that in distilled water (2.543 ± 0.417 × 10^− 2^ mm^3^) (*P*<0.001). Regression lines for wear volume loss as a function of initial roughness in two mediums are illustrated in Fig. 4. The linear regression slope coefficients, standard errors, and *P*-values of the linear regression model are summarized in Table 4. It could be seen that wear volume loss in distilled water showed an increasing trend with the surface roughness. The slope of the regression lines for distilled water was significantly different from zero (*P* < 0.05), which indicated that the increase in roughness tended to have an effect on wear loss in distilled water. However, for the citric acid group, wear loss showed only a slight increase with Ra value but without significant difference. This indicates that the increasing initial roughness had no influence on wear loss of specimen in citric acid.

**Table 4:** Linear regression slope coefficients, standard errors (SE) and F, *P*-values of the linear regression model for wear volume loss in each lubricant

Lubricant	Regression slope (SE)	F	*P*
Distilled water	6.101(0.962)	40.185	<0.001
Citric acid	0.888 (0.595)	2.229	0.166

### Wear morphology

**Fig. 5:**
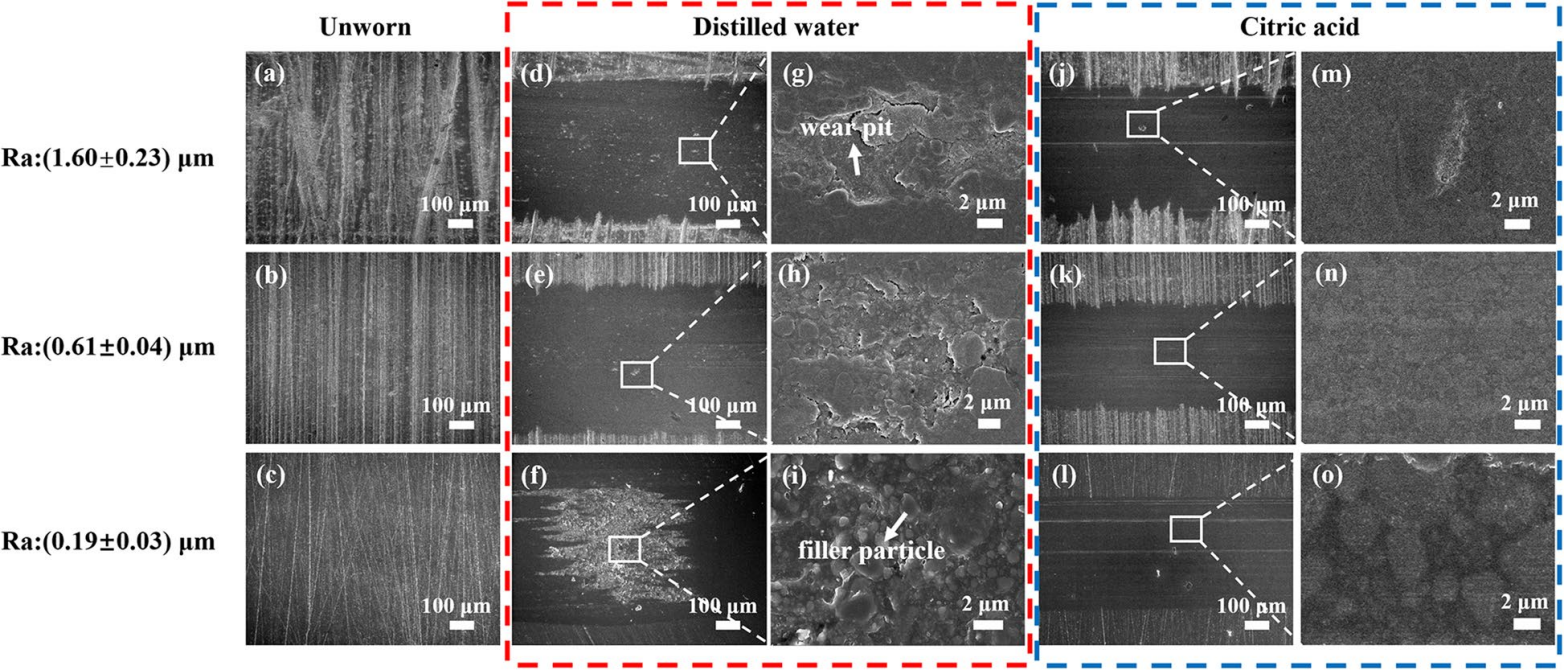
Representative SEM images of the unworn area (the first column on the left) and wear scar on resin composites surface in distilled water and citric acid (inside the red and blue dashed squares, respectively; columns from left at 100X; 5000X magnification)

SEM images of the unworn surfaces are shown in Fig. 5a-c, which exhibited less scratches with the decrease of Ra value. As displayed in Fig. 5d-o, the dimension of the wear scar was wider in distilled water than in citric acid. In distilled water group, severe delamination of the matrix appeared on the scar surface. Small pits (Fig. 5g) and partially exposed filler particles (Fig. 5i) could be observed in wear scar. For the samples in citric acid, shallow ploughing (Fig. 5m) and slight scratching (Fig. 5n and o) could be seen on the surface, which was parallel to the sliding direction.

### Running-in time

**Fig. 6:**
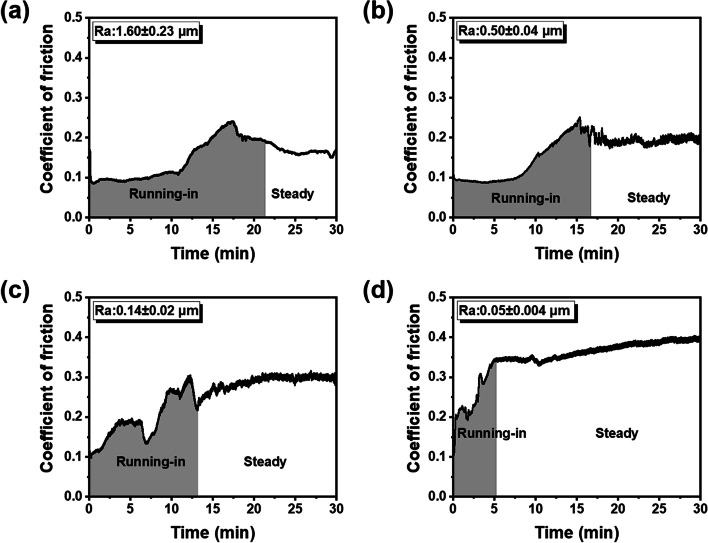
The duration of running-in period for the resin composites with different Ra in distilled water: **a** 1.60 ± 0.23 μm; **b** 0.50 ± 0.04 μm; **c** 0.14 ± 0.02 μm; **d** 0.05 ± 0.004 μm. The gray shadow indicates the running-in stage and the white part indicates the steady stage

**Fig. 7:**
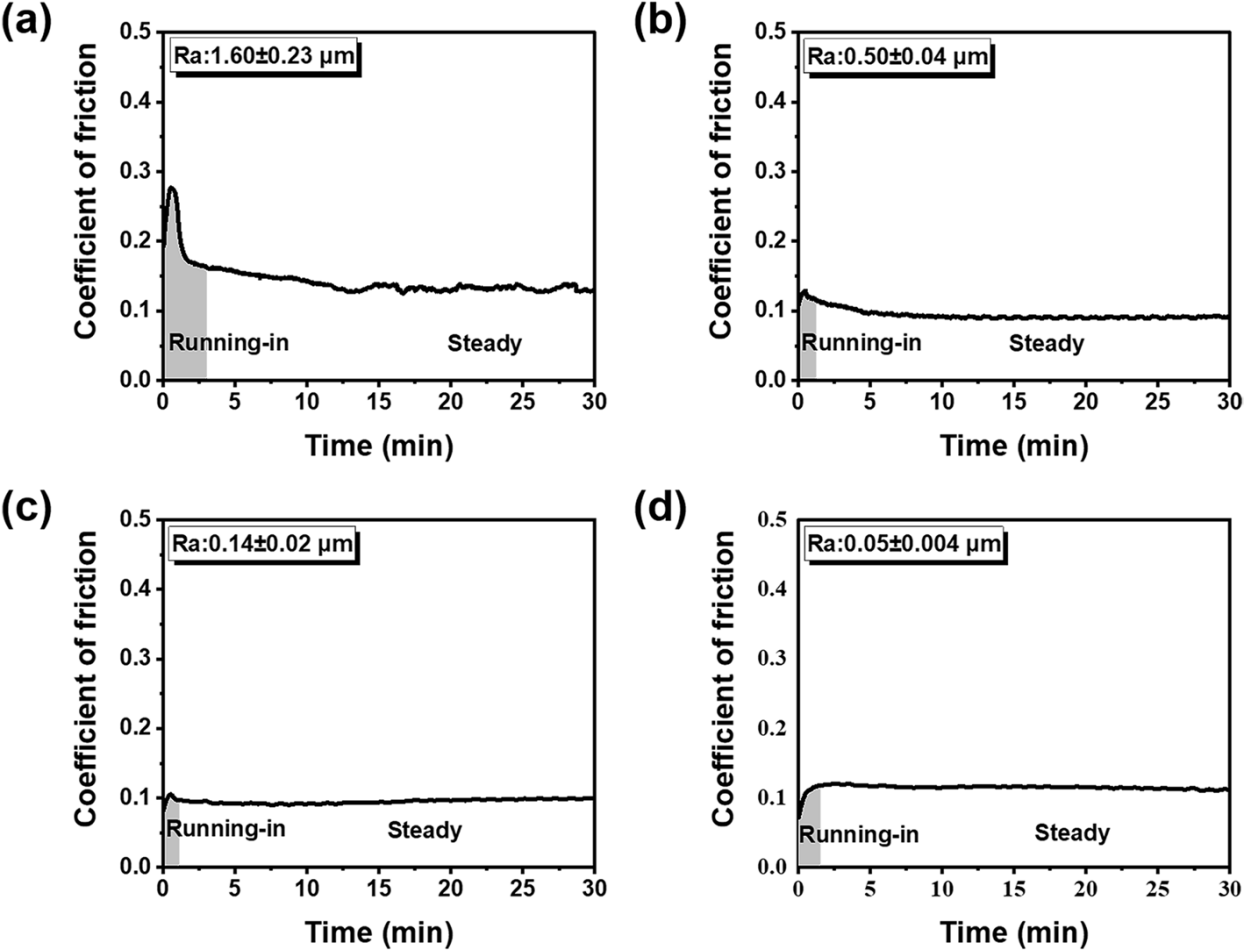
The duration of running-in period for the resin composites with different Ra in citric acid: **a** 1.60 ± 0.23 μm; **b** 0.50 ± 0.04 μm; **c** 0.14 ± 0.02 μm; **d** 0.05 ± 0.004 μm. The gray shadow indicates the running-in stage and the white part indicates the steady stage

**Fig. 8:**
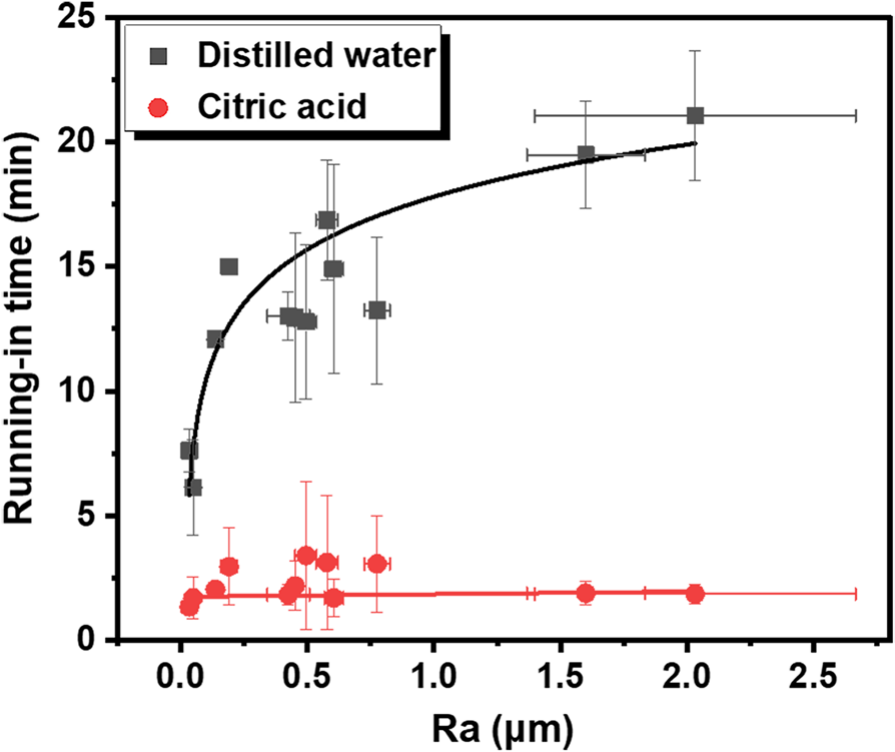
Running-in time as a function of Ra values in distilled water and citric acid. The curves represent the best fit to the experimental data

Figure 6 depicts the details of the running-in period of four representative composite discs in distilled water. The duration of the running-in stage is related to the initial roughness. A rough surface means a longer run-in time. For the sample with the Ra of 1.60 μm (Fig. 6a), the running-in period lasted for 20 min. While for the relatively smooth disk, the COF reached the steady stage earlier (Fig. 6b and c). As the Ra decreased to 0.05 μm (Fig. 6d), the running-in time was only a quarter of that measured in a high roughness sample. Figure 7 shows the representative COF curves of resin composite in acidic medium. It could be seen that the running-in time significantly decreased in citric acid. Specimens with different initial roughness showed a similar running-in time (Fig. 7a-d). Durations of the running-in period versus Ra values in each medium are plotted in Fig. 8. Running-in time showed logarithmic growth with Ra values in distilled water (R^2^ = 0.81), but remained nearly constant in citric acid. For the specimens in distilled water, the duration of the running-in stage increased rapidly from 4.78 to 17.86 min before Ra reached 0.61 μm, then the running-in time grew slowly with the increase of roughness.

## Discussion

Rough surface of resin composite can be a negative factor for the durability of dental restorations. Not only may the irregular surfaces lead to plaque retention [27] and discoloration [28], but the composite restoration also experience severe abrasion, and thereby, shortening the service life of the restoration [29]. In the current study, the relationships between the wear behavior of resin composite and its surface roughness in both citric acid medium and distilled water were described. The results showed that the resin composite exhibited a low COF and less wear loss in citric acid even with high surface roughness, which indicated the potential lubricating effect of citric acid medium. Thus, the null hypothesis that no difference would be found between the wear behavior of composite material in two mediums can be rejected.

In distilled water, resin composite with high surface roughness trends to demonstrate a severe wear behavior. As shown in Figs. 4 and 8, wear loss and running-in time showed increasing trends along with Ra value. Given that the average COF was independent of Ra value (Fig. 2d), the great volume loss found in specimens with high roughness can be attributed to the longer duration of the running-in period. The material loss increases rapidly, also known as high wear rate, during the running-in stage owing to high contact stress by the asperities of rough surfaces [26]. As the wear proceeds, the contact area between the antagonist and sample increases gradually, which reduces the contact pressure and hence the wear rate. Therefore, the duration of the running-in period is critical to the wear performance of composite restoration, and most material loss occurs in this period [30]. The larger the initial roughness value, the longer duration of the running-in stage, which in turn leads to more volume loss of dental composite.

However, regardless of the surface roughness, the resin composite in citric acid exhibited lower COF and shorter running-in time compared to that measured in distilled water, which meant that the citric acid medium reduced the friction of composite material. In fact, the wear volume loss and running-in time of the composite discs in citric acid appeared to be independent of the surface roughness (Figs. 4 and 8), which indicated that the adverse effect of rough surface on wear behavior could be offset by the lubrication action of acids. This can be verified by the microstructure of worn area. For the specimen in acidic lubricant, the wear scar showed shallow ploughing grooves parallel to the sliding direction (Fig. 5m, n and o), while lots of pits and exposed filler particles were observed on the wear scar in distilled water (Fig. 5g and i).

Research has shown that an appropriate concentration of carboxylic acid could significantly shorten the running-in stage and reduce the wear and friction of silicon nitride ceramic/glass contact [31]. It is consistent with the results of the present study, that the friction coefficient and wear volume loss of each disc under citric acid were decreased compared to the values measured in distilled water. During the running-in period, citric acid molecule with its hydrophilic carboxyl group can physically or chemically adsorb onto the surface of the material due to reciprocating friction and temperature rise of microenvironment. Subsequently, a thin mono or multilayer boundary lubricating layer can be formed, which prevents the contact between the two sliding surfaces, thus avoiding the asperity contact and reducing the lateral stress [19, 32]. Therefore, this may be the mechanism for lubricating and anti-wear property derived from the citric acid.

It is important to note that the duration of the running-in stage shows a non-linear relationship with the surface roughness in distilled water (Fig. 8). The running-in time increased rapidly from 4.78 to 17.86 min before Ra reached 0.605 μm, then plateaued at a constant value around 20 min. Therefore, followed by occlusal adjustment and surface degradation, polishing and repolishing procedure are important to reduce running-in period and friction of composite material. According to the findings of the current study, inadequate polishing has little effect on the running-in time unless the Ra value of resin composite reduce to 0.605 μm or below. A more effective polishing result can be achieved only when the Ra value is low enough. However, if a composite restoration with rough surface subject to lateral stress and citric acid condition simultaneously, the duration of running-in can be shortened leading to the low volume of wear loss, which is resulted from the lubricating property of citric acid medium [31].

Citric acid was selected as an acidic medium because it is a natural ingredient of fruit, and also present as an additive in the juices and carbonated beverages, which is commonly seen in daily life and widely used in studies of enamel and dental material wear [9, 33]. According to a randomized controlled trial, the salivary pH ranges 4.1 to 6.0 due to the buffering capacity of the saliva after consumption of different fruit juices. And it takes around 30–60 min for the pH vales to return to baseline [34]. Hence, the pH value of the citric acid and the duration of wear test used in current study are covered by the situation mentioned above. The combination of mechanical and chemical factors allows for better reflection of the oral wear. The current results demonstrated that citric acid medium could reduce the wear loss and coefficient of friction, and alleviated the adverse effects of rough surface on wear behavior. A similar wear reduction results tested under citric acid condition was reported by Figueiredo-Pina et al. [22]. Therefore, if subjected to the mechanical abrasion and citric acid simultaneously, such as eating fruit in daily life, the resin composite restoration may exhibit a mild wear process, which is contrary to what happens with strong acids. These results may provide an opportunity to understanding the wear performance of composite restoration during chewing.

In fact, the impact of acidic environments on the wear resistance of the material depends on the types of acids. Hydrochloric acid (HCl) medium mainly resulted from gastroesophageal reflux disease may only demonstrate erosion effect, causing both bulk loss of resin matrix and surface softening [35]. The softened superficial layer structure appears to be more susceptible to mechanical forces, thus leading to sever wear loss [36]. Without the hydrophilic carboxyl group, hydrochloric acid molecule is unable to adsorb onto the surface of the material. Thus, it’s impossible to form the boundary lubrication layer. Branco et al. in an in vitro comparative study, found that composite material exposed to hydrochloric acid showed higher wear rate than those exposed to saliva and lactic acid [37].

Although citric acid medium exhibits excellent lubricating property, the rough surface of composite restoration still needs to be repolished to obtain a mild wear behavior [38]. Because the citric acid condition, such as consumption of fruits and beverages, is temporary in oral cavity. Once the boundary lubrication layer is worn or rinse off, severe wear process, including high COF and great wear loss, induced by rough surface will recurrence [21]. A ball-on-flat wear model was employed to investigate the wear process. The use of zirconia-on-composite sliding contact mode and the medium with clinically relevant pH allows a better connection to the intraoral scenario. This test model is an effective method to estimate the influence of surface conditions on the wear process of the restorative material. However, future works are needed to consider more factors that may affect the wear performance, including the pH value of citric acid, resin matrix and types of fill particles, which helps to provide a systematic understanding of the wear behavior of resin composite during mastication. Therefore, extrapolation of data in vitro results to the clinical situation has to be done very carefully since the limitation of the current study mentioned above.

## Conclusion

The surface roughness of resin composite mainly affects its running-in stage but has little influence on the steady stage. In distilled water, the higher initial roughness, the longer the running-in stage, which eventually leads to a significant increase in material loss. However, the synergistic relationship between rough surface and citric acid, leading to sever wear process, is not found. On the contrary, citric acid medium can shorten the running-in stage of resin composite to obtain a favorable lubrication and wear reduction performance.

## Data Availability

The datasets used and/or analysed during the current study available from the corresponding author on reasonable request.

## Abbreviations

PICN: Polymer infiltrated ceramic network
COF: Coefficient of friction
SEM: Scanning electron microscopy
Bis-GMA: Bisphenol-A-glycidyl dimethacrylate
Bis-EMA: Ethoxylated bisphenol-A-dimethacrylate
TEGDMA: Triethylene glycol dimethacrylate
UDMA: Urethane dimethacrylate
PEGDMA: Poly (ethylene glycol) dimethacrylate
